# Supplementary material for: Coordination between nucleotide excision repair and specialized polymerase DnaE2 action enables DNA damage survival in non-replicating bacteria
Source: eLife. 2021 Apr 15;10:e67552. doi: 10.7554/eLife.67552 (PMC8102061; doi:10.7554/eLife.67552)
Supplement: Supplementary file 1. [file elife-67552-supp1.docx]

**Supplementary file 1: Strains**

| **Strain name** | **Genotype** | **Strain construction** |
| --- | --- | --- |
| CB15N | NA1000 |  |
| NABC2 | *CB15N; ∆recA* | (Modell et al., 2014) |
| NABC29 | *CB15N; ∆dnaE2* | CB15N was transformed with pNABC148 plasmid to generate deletion of *dnaE2* through two-step recombination procedure (Skerker et al., 2005). |
| NABC149 | *CB15N;dnaE2 (D377A;D379A)* | CB15N was transformed with pNABC132 plasmid to generate specific mutations in the catalytic domain of *dnaE2* through two-step recombination procedure. |
| NABC413  NABC439 | P*_lacI_*-*lacI* (*hfA* locus); P*_lac_*-*dnaA* (*dnaA* locus)  *CB15N; ∆recN* | (Badrinarayanan et al., 2015)  (Badrinarayanan et al., 2015) |
| NABC190 | P*_lacI_*-*lacI* (*hfA* locus); P*_lac_*-*dnaA* (*dnaA* locus); *dnaN*-*YFP*::*spec^R^* | NABC413 was transformed with pNABC198 plasmid to integrate *dnaN-YFP* linked to *spec^R^* at endogenous locus. |
| NABC191 | P*_lacI_*-*lacI* (*hfA* locus); P*_lac_*-*dnaA* (*dnaA* locus); *dnaE*-*mNeonGreen*::*spec^R^* | NABC413 was transformed with pNABC199 plasmid to integrate *dnaE-mNeonGreen* linked to *spec^R^* at endogenous locus. |
| NABC193 | P*_lacI_*-*lacI* (*hfA* locus); P*_lac_*-*dnaA* (*dnaA* locus); *holB*-YFP::*spec^R^* | NABC413 was transformed with pNABC188 plasmid to integrate *holB-YFP* linked to *spec^R^* at endogenous locus. |
| NABC206 | P*_lacI_*-*lacI* (*hfA* locus); P*_lac_*-*dnaA* (*dnaA* locus); *∆dnaE2* | NABC413 was transformed with pNABC148 plasmid to generate deletion of *dnaE2* through two-step recombination procedure. |
| NABC215 | P*_lacI_*-*lacI* (*hfA* locus); P*_lac_*-*dnaA* (*dnaA* locus); *∆dnaE2*; *dnaE*-*mNeonGreen*::*spec^R^* | NABC206 strain was transformed with pNABC199 plasmid to integrate *dnaE-mNeonGreen* linked to *spec^R^* at the endogenous locus. |
| NABC216 | P*_lacI_*-*lacI* (*hfA* locus); P*_lac_*-*dnaA* (*dnaA* locus); *∆dnaE2*; *dnaN-YFP*::spec*^R^* | NABC206 strain was transformed with pNABC198 plasmid to integrate *dnaN-YFP* linked to *spec^R^* at the endogenous locus. |
| NABC217 | P*_lacI_*-*lacI* (*hfA* locus); P*_lac_*-*dnaA* (*dnaA* locus); *∆dnaE2*; P*_xyl_*-*ssb*-*YFP*::*kan^R^* | NABC206 strain was transformed with pNABC419 plasmid to integrate *ssb-YFP* linked to *kan^R^* under the P*_xyl_* locus. |
| NABC218 | P*_lacI_*-*lacI* (*hfA* locus); P*_lac_*-*dnaA* (*dnaA* locus); P_xyl_-*ssb-YFP*::*kan^R^* | NABC413 was transformed with pNABC419 plasmid to integrate *ssb-YFP* linked to *kan^R^* under P*_xyl_* promoter. |
| NABC240 | P*_lacI_*-*lacI* (*hfA* locus); P*_lac_*-*dnaA* (*dnaA* locus); *∆uvrA* | NABC413 was transformed with pNABC417 plasmid to generate deletion of *uvrA* through two-step recombination procedure. |
| NABC247 | P*_lacI_*-*lacI* (*hfA* locus); P*_lac_*-*dnaA* (*dnaA* locus); P_xyl_-*ssb-GFP*::*spec^R^*; *dnaN*-*mCherry*::*kan^R^* | NABC413 was transformed with pNABC415 plasmid to integrate *ssb-GFP* linked to *spec^R^* under P*_xyl_* promoter. Resultant strain was transduced with lysate of a CB15N strain with *dnaN-mCherry* linked to *kan^R^* integrated at the endogenous locus. |
| NABC263 | P*_lacI_*-*lacI* (*hfA* locus); P*_lac_*-*dnaA* (*dnaA* locus); *dnaE2-3X-flag*::*spec^R^* | NABC413 strain transformed with pNABC273 plasmid to integrate *dnaE2-3X-flag* linked to *spec^R^* at the endogenous locus. |
| NABC271 | *P_lacI_-lacI (hfA locus); P_lac_-dnaA (dnaA locus); ∆uvrA; P_xyl_-dnaN-YFP::spec^R^* | NABC240 strain was transformed with pNABC418 plasmid to integrate *dnaN-YFP* linked to *spec^R^* at the P*_xyl_* locus. |
| NABC272 | P*_lacI_*-*lacI* (*hfA* locus); P*_lac_*-*dnaA* (*dnaA* locus); P*_xyl_*-*dnaN-YFP*::*spec^R^* | NABC413 was transformed with pNABC418 plasmid to integrate *dnaN-YFP* linked to *spec^R^* under P*_xyl_* promoter. |
| NABC295A | P*_lacI_*-*lacI* (*hfA* locus); P*_lac_*-*dnaA* (*dnaA* locus); P*_sidA_*-*YFP*::*kan^R^* | NABC413 strain was transformed with pNABC420 plasmid to integrate P*_sidA_*-YFP at the *xyl* locus. |
| NABC295 | P*_lacI_*-*lacI* (*hfA* locus); P*_lac_*-*dnaA* (*dnaA* locus); *dnaE2 (D377A;D379A)*; *dnaN-YFP*::spec*^R^* | NABC190 strain was transformed with pNABC132 plasmid to generate specific mutations in the catalytic domain of *dnaE2* through two-step recombination procedure. |
| NABC356 | P*_lacI_*-*lacI* (*hfA* locus); P*_lac_*-*dnaA* (*dnaA* locus); *∆uvrA*; P*_xyl_*-*ssb*-*YFP*::*kan^R^* | NABC240 strain was transformed with pNABC419 to integrate *ssb-YFP* linked to *kan^R^* under P*_xyl_* promoter. |
| NABC357 | P*_lacI_*-*lacI* (*hfA* locus); P*_lac_*-*dnaA* (*dnaA* locus); *∆mutL*; *dnaN-mCherry*::*kan^R^* | NABC413 was transformed with pNABC416 plasmid to generate deletion of *mutL* through two-step recombination procedure. Resultant strain was transduced with lysate of a CB15N strain with *dnaN-mCherry* linked to *kan^R^* integrated at the endogenous locus. |
| NABC400 | P*_lacI_*-*lacI* (*hfA* locus); P*_lac_*-*dnaA* (*dnaA* locus); *∆uvrA*; P*_sidA_*-*YFP*::*kan^R^* | NABC240 strain was transformed with pNABC420 plasmid to integrate P*_sidA_*-*YFP* at the *xyl* locus. |
| NABC414  NABC435 | P*_lacI_*-*lacI* (*hfA* locus); P*_lac_*-*dnaA* (*dnaA* locus); *∆recA*::kan*^R^*;*dnaN-YFP*::spec*^R^*  P*_lacI_*-*lacI* (*hfA* locus); P*_lac_*-*dnaA* (*dnaA* locus); *∆imuB* | NABC190 strain was transduced with lysate of a strain harboring *recA* deletion and selected for *kan^R^* that is linked to deletion.  NABC413 was transformed with pNABC438 plasmid to generate deletion of *imuB* through two-step recombination procedure. |
| NABC436 | P*_lacI_*-*lacI* (*hfA* locus); P*_lac_*-*dnaA* (*dnaA* locus); *∆imuB*; *dnaN-YFP*::spec*^R^* | NABC435 strain was transformed with pNABC198 plasmid to integrate *dnaN-YFP* linked to *spec^R^* at the endogenous locus. |
| NABC437 | P*_lacI_*-*lacI* (*hfA* locus); P*_lac_*-*dnaA* (*dnaA* locus); *∆recN*::gent*^R^*; *dnaN-YFP*::spec*^R^* | NABC190 strain was transduced with lysate of a strain harboring *recN* deletion (NABC439) and selected for *gent^R^* that is linked to deletion. |

**References**

Badrinarayanan, A., Le, T. B. K., & Laub, M. T. (2015). Rapid pairing and resegregation of distant homologous loci enables double-strand break repair in bacteria. *The Journal of Cell Biology*, *210*(3), 385–400. https://doi.org/10.1083/jcb.201505019

Modell, J. W., Kambara, T. K., Perchuk, B. S., & Laub, M. T. (2014). A DNA damage-induced, SOS-independent checkpoint regulates cell division in Caulobacter crescentus. *PLoS Biology*, *12*(10), e1001977. https://doi.org/10.1371/journal.pbio.1001977

Skerker, J. M., Prasol, M. S., Perchuk, B. S., Biondi, E. G., & Laub, M. T. (2005). Two-Component Signal Transduction Pathways Regulating Growth and Cell Cycle Progression in a Bacterium: A System-Level Analysis. *PLoS Biology*, *3*(10), e334. https://doi.org/10.1371/journal.pbio.0030334
