# Supplementary material for: Coordination between nucleotide excision repair and specialized polymerase DnaE2 action enables DNA damage survival in non-replicating bacteria
Source: eLife. 2021 Apr 15;10:e67552. doi: 10.7554/eLife.67552 (PMC8102061; doi:10.7554/eLife.67552)
Supplement: Supplementary file 2. [file elife-67552-supp2.docx]

**Supplementary file 2: Plasmids**

| **Plasmid name** | **Construct details** | | **Antibiotic marker** |  |
| --- | --- | --- | --- | --- |
|  | |  |  |  |
| pNPTS138 | | (Skerker et al., 2005) | Kanamycin |  |
| pMCS1 | | (Thanbichler et al., 2007) | Spectinomycin |  |
| pYFPC1 | | (Thanbichler et al., 2007) | Spectinomycin |  |
| pXGFPC1 | | (Thanbichler et al., 2007) | Spectinomycin |  |
| pXYFPC1 | | (Thanbichler et al., 2007) | Spectinomycin |  |
| pXYFPC2 | | (Thanbichler et al., 2007) | Kanamycin |  |
| pNABC132 | | 600 bp internal fragment of *dnaE2* was amplified using AMJ_oligo_006 and AMJ_oligo_007 (reverse primer harboring mutations). Another 600 bp internal fragment of *dnaE2* was amplified using AMJ_oligo_008 (forward primer harboring mutations) and AMJ_oligo_009. These two fragments were assembled with linearized pNPTS138 vector using Gibson assembly. | Kanamycin |  |
| pNABC148 | | 600 bp fragments upstream and downstream of *dnaE2* genomic locus were amplified from *C. crescentus* gDNA using RR_oligo_021/RR_oligo_022 (upstream fragment) and RR_oligo_023/RR_oligo_024 (downstream fragment) primer pairs. These fragments were assembled with BamH1/Nhe1 linearized pNPTS138 vector using Gibson assembly. | Kanamycin |  |
| pNABC188 | | Full length *holB* was amplified from *C. crescentus* gDNA using AMJ_038 and AMJ_039, restriction digested with Nde1/Kpn1 and ligated to Nde1/Kpn1 digested pYFPC1 vector. *holB* and *YFP* in this construct was separated by 60 bp resulting in a 20 amino acid linker in the fusion protein. | Spectinomycin |  |
| pNABC198 | | Full length *dnaN* was amplified using AB_036 and AB_039, digested with Nde1/EcoR1 and ligated to Nde1/EcoR1 digested pYFPC1 vector. *dnaN* and *YFP* in this construct was separated by 36 bp resulting in a 12 amino acid linker in the fusion protein. | Spectinomycin |  |
| pNABC199 | | C-terminal region of *dnaE* was amplified using AB_oligo_791 and RR_oligo_004, restriction digested with NdeI/KpnI and ligated to NdeI/KpnI digested pYFPC1 plasmid. This region was further amplified from this construct using forward primer (AMJ_oligo_16) with 5' homology for region upstream of Nde1 and reverse primer (AMJ_oligo_17) with 5' homology for the region downstream of Kpn1 site in pMCS1 vector. Similarly, mNeonGreen fragment was amplified using forward primer (AMJ_oligo_18) with 5' homology for region downstream of Kpn1 and reverse primer (AMJ_oligo_15) with 5' homology downstream of Nhe1 site in pMCS1 vector. These amplicons and Nde1/Nhe1 digested pMCS1 vector were assembled using Gibson assembly to generate a construct where *dnaE* C-terminal was cloned in frame with *mNeonGreen*, separated by 60 bp resulting in a 20 amino acid linker in the fusion protein. | Spectinomycin |  |
| pNABC273 | | C-terminal region of *dnaE2* was amplified using RR_oligo_003 and IS_oligo_047 (harboring 5' overhangs for 3X-flag sequence), digested with Nde1/Nhe1, and ligated to Nde1/Nhe1 digested pMCS1 vector. | Spectinomycin |  |
| pNABC415 | | *ssb* fragment from pNABC419 was retrieved by Nde1/Kpn1 digestion and ligated to Nde1/Kpn1 digested pXGFPC1 plasmid. | Spectinomycin |  |
| pNABC416 | | 600 bp fragments upstream and downstream of *mutL* genomic locus were amplified from *C. crescentus* gDNA using PS_oligo_049/AMJ_oligo_061 (upstream fragment) and AMJ_oligo_062/PS_oligo_054 (downstream fragment) primer pairs. These fragments were assembled with linearized pNPTS138 vector using Gibson assembly. | Kanamycin |  |
| pNABC417 | | 600 bp fragments upstream and downstream of *uvrA* genomic locus were amplified from *C. crescentus* gDNA using PS_oligo_037/AMJ_oligo_057 (upstream fragment) and AMJ_oligo_058/PS_oligo_042 (downstream fragment) primer pairs. These fragments were assembled with BamH1/Nhe1 linearized pNPTS138 vector using Gibson assembly. | Kanamycin |  |
| pNABC418 | | *dnaN-YFP* fragment from pNABC198 construct was retrieved by restriction digestion with Nde1/Nhe1 and ligated to Nde1/Nhe1 digested pXYFPC1 plasmid. | Spectinomycin |  |
| pNABC419 | | Full length *ssb* amplified was using AB_oligo_658 and AB_oligo_659, digested with Nde1/Kpn1 and ligated with Nde1/Kpn1 digested pXYFPC2. *ssb* and *YFP* in this construct was separated by 60 bp resulting in a 20 amino acid linker in the fusion protein. | Kanamycin |  |
| pNABC420  pNABC438 | | pXYFPC2 vector was amplified using AB_oligo_651 and AB_oligo_652 and P*_sidA_*-*YFP* fragment was amplified from a replicating plasmid habouring YFP under P*_sidA_* promoter using AC_oligo_322 and AC_oligo_321. The vector and insert fragments were assembled with Gibson assembly.  600 bp fragments upstream and downstream of *imuB* genomic locus were amplified from *C. crescentus* gDNA using RR_oligo_017/RR_oligo_018 (upstream fragment) and RR_oligo_019/RR_oligo_020 (downstream fragment) primer pairs. These fragments were assembled with linearized pNPTS138 vector using Gibson assembly. | Kanamycin  Kanamycin |  |

**References**

Skerker, J. M., Prasol, M. S., Perchuk, B. S., Biondi, E. G., & Laub, M. T. (2005). Two-Component Signal Transduction Pathways Regulating Growth and Cell Cycle Progression in a Bacterium: A System-Level Analysis. *PLoS Biology*, *3*(10), e334. https://doi.org/10.1371/journal.pbio.0030334

Thanbichler, M., Iniesta, A. A., & Shapiro, L. (2007). A comprehensive set of plasmids for vanillate- and xylose-inducible gene expression in Caulobacter crescentus. *Nucleic Acids Research*, *35*(20), e137. https://doi.org/10.1093/nar/gkm818
