# Supplementary material for: Coordination between nucleotide excision repair and specialized polymerase DnaE2 action enables DNA damage survival in non-replicating bacteria
Source: eLife. 2021 Apr 15;10:e67552. doi: 10.7554/eLife.67552 (PMC8102061; doi:10.7554/eLife.67552)
Supplement: Supplementary file 3. [file elife-67552-supp3.docx]

**Supplementary file 3: Oligos**

| **Primer name** | **Sequence** |
| --- | --- |
| AB_oligo_036 | TTATCATATGAAGCTTACGATCGAACGGGCGGCG |
| AB_oligo_039 | TTATGAATTCCGGACCCGCAGCGGCATCAGCAC |
| AB_oligo_651 | CTGGACCTCTTGCCCATGACCGA |
| AB_oligo_652 | GCTAGCTGCAGCCCGGGGG |
| AB_oligo_658 | TTATCATATGGCTGGCAGCGTCAACAAGG |
| AB_oligo_659 | TTATGGTACCGAACGGGATCTCGTCGTCCAGATC |
| AB_oligo_791 | TTATCATATGGATCGCCCACGCCCAGAG |
| AC_oligo_321 | AACTAGTGGATCCCCCGGGCTGCAGCTAGCTTACTTGTACAGCTCGTCCATGCCGA |
| AC_oligo_322 | GGTCAGGTCGGTCATGGGCAAGAGGTCCAGCACCCGCCCATCACCCACAGATGC |
| AMJ_oligo_006 | CAAGCTTCTCTGCAGGATATCTGAGAGTCTCGGTCGGACCTCCGGCGCG |
| AMJ_oligo_007 | GAAGTCGACGGCGATGGCGGGCGGCTCG |
| AMJ_oligo_008 | CGAGCCGCCCGCCATCGCCGTCGACTTC |
| AMJ_oligo_009 | CGGAGACGCGTCACGGCCGAAGCGGCTCTCGACCTGGAACACGCCTACG |
| AMJ_oligo_015 | GAACTAGTGGATCCCCCGGGCTGCAGCTAGTTACGAATTCGAGCTCGCGCTGCC |
| AMJ_oligo_016 | TGCTTAATGAATTACAACAGTTTTTATATAGATCGCCCACGCCCAGAG |
| AMJ_oligo_017 | GGTGGCCGACCGGTGACGCGTAACGTTCGAATTCTCCGGAGCTCGAGATCTTAAGGTACCAACGTCTTCCAGCAGCGCCAC |
| AMJ_oligo_018 | GGTACCTTAAGATCTCGAGCTCCGGAGAATTCGAACGTTACGCGTCACCGGTCGGCCACCATGGCAAGCCTGCCGGCGAC |
| AMJ_oligo_038 | TTATCATATGGACAAGCAGGCCGCCGAAGCCCTGA |
| AMJ_oligo_039 | TTATGGTACCCAGGCCCAGTCGGCGGGCGC |
| AMJ_oligo_057 | GCCTGCTGAGCCGCCTTAGTTTTCCGGAACGTTGGAC |
| AMJ_oligo_058 | GTCCAACGTTCCGGAAAACTAAGGCGGCTCAGCAGGC |
| AMJ_oligo_061 | TAGGGGGCGCTCTGGCCTCACATCAAGCGGACTTTCACGGG |
| AMJ_oligo_062 | CCCGTGAAAGTCCGCTTGATGTGAGGCCAGAGCGCCCCCTA |
| IS_oligo_047 | TTATGCTAGCTTACTTATCGTCATCGTCTTTGTAATCAATATCATGATCCTTGTAGTCTCCGTCGTGGTCCTTATAGTCCACCATGGTGGCCGACCGGTG |
| PS_oligo_037 | CAAGCTTCTCTGCAGGATATCTGCTTGGCGATGGCGTCACCT |
| PS_oligo_042 | CGGAGACGCGTCACGGCCGAAGTCTACGCAGACGTGGATCTTG |
| PS_oligo_049 | CAAGCTTCTCTGCAGGATATCTGGTCAAATGCTTCTCCAGCCG |
| PS_oligo_054 | CGGAGACGCGTCACGGCCGAAGGAAGGAGACGAGACGATGGA |
| RR_oligo_003 | TTATCATATGAACTTCTGGCCGAGGCCGAC |
| RR_oligo_004  RR_oligo_017  RR_oligo_018  RR_oligo_019  RR_oligo_020 | TTATGGTACCAACGTCTTCCAGCAGCGCCAC  CAAGCTTCTCTGCAGGATATCTGTGTCGCTGCTGGAGGACGC  GATTTGAGCCCCCTACATAGACCGAGCCTGATCATCGACG  CAGGCTCGGTCTATGTAGGGGGCTCAAATCCTCCCCC  CGGAGACGCGTCACGGCCGAAGCATAGGCCCGACTGGCGGC |
| RR_oligo_021 | CAAGCTTCTCTGCAGGATATCTGGACGCTGGCGCCGTTGATC |
| RR_oligo_022 | ATCGCGCCCCGCTCACATGTTAGGTCCTCCCCCTCGC |
| RR_oligo_023 | GGAGGACCTAACATGTGAGCGGGGCGCGATCCT |
| RR_oligo_024 | CGGAGACGCGTCACGGCCGAAGGCGACATGCGGGTCAGCA |
