# Supplementary material for: Coordination between nucleotide excision repair and specialized polymerase DnaE2 action enables DNA damage survival in non-replicating bacteria
Source: eLife. 2021 Apr 15;10:e67552. doi: 10.7554/eLife.67552 (PMC8102061; doi:10.7554/eLife.67552)
Supplement: Supplementary file 4. [file elife-67552-supp4.docx]

**Supplementary file 4: exact p-values**

| **Figure** | **Unpaired t-test** | **p-value** | |
| --- | --- | --- | --- |
| Figure 4-figure | 0.125 μg/ml MMC, wild type - 0 min vs. 90 min | 0.0002 | *** |
| supplement 1C | 0.25 μg/ml MMC, wild type - 0 min vs. 90 min | 0.0004 | *** |
|  | 0.5 μg/ml MMC, wild type - 0 min vs. 90 min | 0.0076 | ** |
|  | 0.75 μg/ml MMC, wild type - 0 min vs. 90 min | 0.0006 | *** |
|  | 0.125 μg/ml MMC, *ΔdnaE2* - 0 min vs. 90 min | 0.0115 | * |
|  | 0.25 μg/ml MMC, *ΔdnaE2* - 0 min vs. 90 min | 0.0129 | * |
|  | 0.5 μg/ml MMC, *ΔdnaE2* - 0 min vs. 90 min | 0.9199 | ns |
|  | 0.75 μg/ml MMC, *ΔdnaE2* - 0 min vs. 90 min | 0.1303 | ns |
| Figure 4-figure | 75 J/m^2^ UV, 90 min recovery - wild type vs. *ΔdnaE2* | 0.0052 | ** |
| supplement 1D | 150 J/m^2^ UV, 90 min recovery - wild type vs. *ΔdnaE2* | 0.0028 | ** |
| Figure 4-figure | wild type, no recovery - control vs. MMC | 0.0177 | * |
| supplement 1G | wild type, recovery - control vs. MMC | 0.0025 | ** |
|  | ΔdnaE2, no recovery - control vs. MMC | 0.7278 | ns |
|  | *ΔdnaE2*, recovery - control vs. MMC | 0.761 | ns |
| Figure 5D | 0.125 μg/ml MMC, wild type - recovery vs. no recovery | 0.09 | ns |
|  | 0.125 μg/ml MMC, *ΔdnaE2* - recovery vs. no recovery | 0.2143 | ns |
|  | 0.25 μg/ml MMC, wild type - recovery vs. no recovery | 0.3478 | ns |
|  | 0.25 μg/ml MMC, *ΔdnaE2* - recovery vs. no recovery | 0.3716 | ns |
|  | 0.5 μg/ml MMC, wild type - recovery vs. no recovery | 0.0021 | ** |
|  | 0.5 μg/ml MMC, *ΔdnaE2* - recovery vs. no recovery | 0.3977 | ns |
|  | 0.75 μg/ml MMC, wild type - recovery vs. no recovery | 0.0025 | ** |
